# Psychotropic and anti-dementia treatment in elderly persons with clinical signs of dementia with Lewy bodies: a cross-sectional study in 40 nursing homes in Sweden

**DOI:** 10.1186/s12877-018-0740-4

**Published:** 2018-02-17

**Authors:** Iris Zahirovic, Gustav Torisson, Carina Wattmo, Elisabet Londos

**Affiliations:** 1 Clinical Memory Research Unit, Department of Clinical Sciences, Lund University, Malmö, Sweden; 2 Memory Clinic, Skåne University Hospital, SE-205 02 Malmö, Sweden

**Keywords:** Dementia with Lewy bodies, Antipsychotics, Nursing homes, Elderly, Medication

## Abstract

**Background:**

Elderly persons with a dementia diagnosis often suffer from different neuropsychiatric symptoms (NPS) such as delusions, hallucinations, depression, anxiety, irritability and agitation. Currently, the medical treatment for NPS consists mostly of psychotropic medication such as hypnotics/sedatives, anxiolytics and antipsychotics. In elderly persons with dementia, usage of antipsychotics is less appropriate because of the risk of side effects such as parkinsonism, rapid cognitive decline, cerebrovascular events and finally mortality. Furthermore, elderly persons with dementia with Lewy bodies (DLB) are often hypersensitive to antipsychotics with numerous serious adverse events such as somnolence, sedation, extra-pyramidal symptoms, delirium and increased mortality. The aim of this study was to investigate the usage of psychotropics with a focus on antipsychotics and anti-dementia medication (according to the Anatomical Therapeutic Chemical Classification System) in elderly persons with clinical signs of DLB living in dementia nursing homes (NHs) in Sweden.

**Methods:**

Between 2012 and 2013, we applied a specially designed questionnaire that covered the clinical DLB features according to the consensus criteria of DLB. We also collected computerized medical lists from the Swedish National Medication Dispensing System from the same period. All dementia NHs (*n* = 40) in Malmö, the third largest city in Sweden, were covered. Of 650 eligible residents, 610 (94%) were included with 576 medical lists. The mean age was 86 years and 76% were women.

**Results:**

Treatment with antipsychotics was seen in 22% of residents, hypnotics/sedatives in 41%, antidepressants in 50% and anxiolytics in 58%. We also found an increasing usage of antipsychotics from 25% to 43% in residents with the increasing number of DLB features. Anti-dementia medications were found in 45% of the elderly with a dementia diagnosis. However, residents with two or more DLB features had less anti-dementia medication (37%) than the rest of the dementia-diagnosed NH residents (62–69%).

**Conclusions:**

Residents with 2–4 DLB clinical features in Swedish NHs receive an unfavourable medical treatment with high antipsychotic usage and insufficient anti-dementia medication. These findings show the importance of identifying elderly persons with DLB features more effectively and improving the collaboration with nursing care to provide better medical prescription.

## Background

According to the Delphi consensus study on the global prevalence of dementia in 2005, there were 24.3 million people with dementia, with a predicted doubling every 20 years [1, 2]. Dementia with Lewy bodies (DLB) is considered the second most common neurodegenerative disorder after Alzheimer’s disease (AD) with both neurological and psychological symptomatology [3, 4]. According to the consensus criteria, the core clinical features of DLB are visual hallucinations, fluctuating cognition, parkinsonism and REM sleep behaviour disorder. At the later stage, elderly persons with DLB as well as other people with dementia often have neuropsychiatric symptoms (NPS) such as delusions hallucinations, depression, agitation, aggression, anxiety and irritability, with prevalence varying between 84% and 90% [5, 6]. The definite DLB diagnosis usually consists of Lewy bodies, Lewy neurites and amyloid plaques in different proportions [7–9]. The relation between DLB signs and DLB pathology is illustrated in a study which showed that the amount of Lewy body pathology correlated to the number of the four core DLB signs [10].

The medical treatment for NPS consists of different psychotropic medications (hypnotics/sedatives, anxiolytics, antipsychotics and anti-depressants), and mostly antipsychotics, even if the effect of both conventional and atypical antipsychotics is still questionable for improvement of some NPS [11, 12]. In patients with dementia, usage of antipsychotics is less appropriate because of the increased risk of nursing home (NH) admission, a more rapid cognitive decline, cerebrovascular events and finally mortality [13–16]. In addition, elderly patients with DLB are hypersensitive to antipsychotics, due to defective upregulation of dopamine 2 receptors, and in the later stage of their disease, they often live in NHs [17–19].

The US Food and Drug Administration (FDA), the European Medicines Agency (EMA) as well as the Swedish National Board of Health and Welfare (NBHW) have promoted the reduction of antipsychotics and other psychotropics and encourage the usage of other pharmacological and non-pharmacological treatments [20–23]. This is mainly because of the risk of numerous and serious adverse events such as parkinsonism, somnolence/sedation, extra-pyramidal symptoms, delirium and finally increased mortality [16].

During the last decade, antipsychotics have been the subject of many different regulatory risk communication methods and black box warnings worldwide [24–26]. However, the usage of psychotropics is still high and according to several Swedish, Danish and Norwegian studies varies greatly (39%–71%). These variations are seen in the elderly population with dementia, among the institutionalized and non-institutionalized elderly and by primary care or specialist clinic medical usage [25, 27, 28].

In a previous study of the same population, we reported an underdiagnosed prevalence of clinical DLB features in NHs [29]. The aim of the present study was to investigate the usage of psychotropic medication (antipsychotics, hypnotics/sedatives, anti-depressants, anxiolytics and anti-dementia medication) in Swedish NHs with a special focus on patients with clinical features of DLB.

## Methods

### Study design

The study material consists of data from the dementia NH residents in Malmö, the third largest Swedish city with over 320,000 inhabitants. To gather accurate information on the actual number of dementia NHs, for the period 2012–2013, we contacted the head account manager for dementia NHs in the city of Malmö. Thereafter, from January 2012 to March 2013, we invited all 40 dementia NHs in Malmö to participate in the study. All residents aged ≥65 years had the same electronic hospital medical record system and updated medical lists during the study period.

### Nursing homes

The NHs in Malmö each housed an average of 15 elderly residents (range 4–45) with a staff consisting of a resident physician (specialist from primary care), 1 or 2 head nurses (with 3 years of university) and a varying number [4–8] of assistant nurses (with 2 years of nursing care school). The elderly residents either already had a dementia diagnosis or were undergoing a dementia investigation procedure. All resident physicians are requested to follow the guidelines for dementia investigation from the Swedish NBHW [30]. The minimal investigation, the so-called “Basal dementia investigation”, consists of cognitive testing (Mini–Mental State Examination, cube and clock drawing tests), relevant laboratory tests, cone beam computed tomography (CBCT) and/or magnetic resonance (MR) imagining (evaluated by a radiologist) and structural anamnestic interview by the doctor (patient and family members).

The elderly person becomes an NH resident after a decision made by a care organizer from the local authority (communal/municipal) following care planning with the elderly person, the family members and a doctor or head nurse who had consulted with the doctor.

### Data collection

The inclusion criterion was all residents living at NHs ≥65 years. The exclusion criterion was residents in a palliative state of treatment. The main clinical outcome measure was a questionnaire that was specially designed to cover clinical signs of DLB according to the consensus criteria from the third report of the DLB Consortium [7] but also compatible with the diagnostic form of DLB Diagnostic Symptoms of the Lewy Body Dementia Association [31]. We decided to choose observable descriptions of possible DLB signs. Prior to entering the study, each NH separately received a specially designed presentation seminar given by a resident physician (IZ) to all nursing staff. For this study, there was also the opportunity of individual medical education, clarification and answering questions from the nursing staff (head nurses and assistant nurses). There were also possibilities for the resident physician to demonstrate to the nursing staff how the clinical features from the questionnaire could be expressed in practice in the elderly (with their verbal consent) living in NHs. The nursing staff were instructed to register the presence/absence of seven actual clinical features at the time of scoring: 1) Parkinson’s disease (PD) or parkinsonism; 2) rigidity, signs of stiffness; 3) tremor and/or a rigid posture and walk; 4) a weak voice; 4) balance problems, dizziness, faints, falling easily and/or more frequently; 5) visual hallucinations; 6) excessive daytime sleepiness, variations in attention and wakefulness; and 7) sleep disorder, acting out dreams during sleep (sometimes violently), shouting out at night, nightmares. A more detailed description of the questionnaires is presented in an earlier publication [29].

### Medical data

During the same period, the head nurse collected actual medical lists from the Swedish National Medication Dispensing System (NMDS) together with the questionnaire. From the computerized hospital medical records, we collected data on dementia diagnoses (yes/no and dementia type). From the medical lists, we categorized the psychotropic medicines according to the Anatomical Therapeutic Chemical (ATC) Classification System: antipsychotics (N05A), anxiolytics (N05B), hypnotics/sedatives (N05C), anti-depressants (N06A) and anti-dementia medicine (N06D) [32].

### Statistical analysis

For the statistical analysis, we used the IBM Statistical Package for the Social Sciences (SPSS) for Windows (version 22.0; IBM Corporation, Armonk, NY, USA). For comparisons of two independent groups, we used the Student’s *t* test for normally distributed data and the Mann–Whitney *U* test for non-parametric data. NH residents were divided into dichotomous groups: dementia (yes/no), DLB signs (0–1 DLB and 2–4 DLB) and age (≤85 and ≥86 years) (Tables 1, 2 and 3). Categorical data were investigated using the chi-squared test. The significance level was set to *P* < 0.05. A Bonferroni correction for multiple testing of the groups was applied by setting the *P*-value at < 0.01.

**Table 1:** Demographics

	Study population N (%)	DLB 0–1^a^ N (%)	DLB 2–4^b^ N (%)	Anti-dementia medication^c^ N (%)	Antipsychotic medication^d^ N (%)
Sex N (%)					
Male	146 (24)	112 (77)	34 (23)	51 (38)	31 (21)
Female	464 (76)	374 (81)	90 (19)	141 (32)	101 (22)
Dementia diagnosis					
Yes N (%)	440 (74)	348 (79)	92 (21)	192 (45)	102 (23)
Different dementia diagnoses^e^					
Alzheimer’s disease	115 (26)	92 (80)	23 (20)	71 (64)	30 (26)
Alzheimer’s mixed	97 (22)	83 (86)	14 (14)	66 (69)	25 (26)
Vascular dementia	85 (19)	69 (81)	16 (19)	5 (6)	20 (24)
DLB/PDD^f^	22 (5)	2 (9)	20 (91)	13 (62)	5 (23)
Dementia NOS^g^	121 (28)	102 (84)	19 (16)	37 (32)	22 (18)

^a^DLB 0–1: none or one clinical feature of dementia with Lewy bodies (DLB)

^b^DLB 2–4: two or more DLB clinical features

^c^Anti-dementia medication (N06D)

^d^Antipsychotic medication (N05A)

^e^Dementia diagnoses found in 440 (100%) residents

^f^DLB/PDD: DLB/Parkinson’s disease with dementia

^g^Dementia NOS: dementia not otherwise specified

**Table 2:** Psychotropic medication

	ATC^a^	Study population^b^ N (%)	Dementia diagnoses^c^		*P*-value	Clinical signs^d^of DLB		*P*-value	Age groups^e^		*P*-value
			Yes N (%)	No N (%)		0–1 N (%)	2–4 N (%)		≤85 years N (%)	≥86 years N (%)	
Antipsychotics											
*Any*	N05A	132 (21.6)	102 (23.2)	29 (18.8)		93 (19.1)	39 (31.5)	*	71 (27.4)	61 (17.4)	**
*Haloperidol*		42 (7.1)	28 (6.6)	14 (9.3)		25 (5.3)	17 (14.3)	**	23 (9.3)	19 (5.5)	
*Risperidone*		57 (9.6)	49 (11.5)	8 (5.3)		45 (9.5)	12 (10.1)		29 (11.7)	28 (8.1)	
*Other* ^f^		11 (1.9)	9 (2.1)	2 (1.3)		7 (1.5)	4 (3.4)		6 (2.4)	5 (1.5)	
Anxiolytics											
*Any*	N05B	336 (58.4)	263 (61.7)	73 (49.0)	*	251 (54.8)	85 (72.6)	***	136 (56.2)	200 (60.1)	
*Oxazepam*		308 (53.6)	248 (58.2)	60 (40.3)	**	228 (49.8)	80 (68.4)	***	125 (51.7)	183 (55.0)	
*Diazepam*		44 (7.7)	31 (7.3)	13 (8.7)		25 (5.5)	19 (16.2)	***	22 (9.1)	22 (6.6)	
Hypnotics/sedatives											
*Any*	N05C	234 (40.7)	176 (41.3)	58 (38.9)		186 (40.6)	48 (41.0)		103 (42.6)	131 (39.3)	
*Zopiclone*		189 (32.9)	139 (32.6)	50 (33.6)		145 (31.7)	44 (37.6)		78 (32.2)	111 (33.3)	
*Other* ^g^		77 (13.4)	67 (15.7)	10 (6.7)	**	68 (14.8)	9 (7.7)	**	39 (16.1)	38 (11.1)	
Anti-depressants											
*Any*	N06A	290 (50.4)	225 (52.8)	65 (43.6)	**	233 (48.4)	57 (60.6)	*	129 (53.3)	161 (48.3)	
*SSRI*		204 (35.5)	156 (36.6)	48 (32.2)		157 (34.3)	47 (40.2)		88 (36.4)	116 (34.8)	
*Mirtazapine*		127 (22.1)	103 (24.2)	24 (16.1)	*	99 (20.6)	28 (29.8)		58 (24.0)	69 (20.7)	
*Venlafaxine*		18 (3.1)	14 (3.3)	4 (2.7)		15 (3.1)	3 (3.2)		13 (5.4)	5 (1.5)	*
*Other*		6 (1.0)	4 (0.9)	2 (1.3)		4 (0.9)	2 (1.3)		2 (0.8)	4 (1.2)	

^a^Anatomical Therapeutic Chemical (ATC) Classification System

^b^All participants: 610 (100%), with registered medical lists for 575 (94%) participants

^c^Dementia diagnoses: there were (*N* = 440) with a dementia diagnosis and (*N* = 154)participants without a dementia diagnosis

^d^Clinical signs: visual hallucinations, fluctuating cognition, parkinsonism and REM sleep behaviour disorder

^e^Age group total 610 (100%): ≤85 years (*N* = 259) and ≥86 years (*N* = 351)

^f^Other (N05A): Clozapine (n = 1), Olanzapine (*n* = 8), Quetiapine (n = 2)

^g^Other (N05C): Melatonine (*n* = 21), Klometiazol (*n* = 58), Nitrazepam (*n* = 6), Propriomazin (n = 5)

*P-value < 0.05, **P-value< 0.005, ***P-value < 0.001

**Table 3:** Anti-dementia medication

	ATC ^a^	Study population^b^ N (%)	Dementia diagnoses^c^		*P*-value	Clinical signs^d^ of DLB		*P*-value	Age groups^e^		*P*-value
			Yes N (%)	No N (%)		0–1^||^ N (%)	2–4^||^ N (%)		≤85 years N (%)	≥86 years N (%)	
Anti-dementia drugs (All)	N06D	192 (33.3)	192 (45.0)	0 (0.0)	***	149 (32.5)	43 (36.8)		104 (43.0)	88 (26.3)	***
ChEI (Any) ^f^	N06DA	123 (21.4)	123 (28.8)	0 (0.0)	***	104 (22.7)	19 (16.2)		67 (27.7)	56 (16.8)	**
*Donepezil*	N06DA	42 (7.3)	42 (9.8)	0 (0.0)	***	41 (8.9)	1 (0.9)	***	19 (7.9)	23 (6.9)	
*Rivastagmin*	N06DA	31 (5.4)	31 (7.3)	0 (0.0)	***	21 (4.6)	10 (8.5)		25 (10.3)	6 (1.8)	***
*Galantamin*	N06DA	50 (8.7)	50 (11.7)	0 (0.0)	***	42 (9.2)	8 (6.8)		23 (9.5)	27 (8.1)	
Memantin	N06DX	100 (17.4)	100 (23.4)	0 (0.0)	***	69 (15.0)	31 (26.5)	**	59 (24.4)	41 (12.3)	***
ChEI & Memantin	N06DA N06DX	31 (5.4)	31 (7.3)	0 (0.0)	***	24 (5.2)	7 (6.0)		22 (9.1)	9 (2.7)	***

^a^Anatomical Therapeutic Chemical (ATC) Classification System

^b^All participants: 610 (100%), with registered medical lists for 575 (94%) participants

^c^Dementia diagnoses: there were (*N* = 440) with a dementia diagnosis and (N = 154)participants without a dementia diagnosis

^d^Clinical signs: visual hallucinations, fluctuating cognition, parkinsonism and REM sleep behaviour disorder

^e^Age group total 610 (100%): ≤85 years (*N* = 259) and ≥86 years (*N* = 351)

^f^ChEI: cholinesterase inhibitor

*P-value < 0.05, **P-value< 0.005, ***P-value < 0.001

## Results

### Study population

In this study, 40 NHs with 650 residents were invited to participate. Of these, 30 residents declined participation and 10 had missing data. Thus, the final study population consisted of 610 elderly residents with collected medical lists for 576 (94%), hospital medical records for 594 (97%) and completed questionnaires for all residents. The mean age was 86 (± 6.9) years and 76% were women; 74% of residents had a dementia diagnosis (Table 1).

The number of different DLB signs, from the questionnaires, were used to create two groups: residents with 0–1 DLB signs and those with 2–4 DLB signs. In an earlier study of the same population, we reported that 84% had 0–1 and 16% had 2–4 main clinical features of DLB [29]. The residents with 2–4 DLB signs had the following dementia diagnoses: AD in 19%, DLB/Parkinson’s disease with dementia (PDD) in 17%, dementia not otherwise specified (NOS) in 16%, vascular dementia (VaD) in 13%, AD mixed dementia (AD-MIX) in 12% and no diagnosis in 23%. Finally, 22 of the residents were already diagnosed with DLB/PDD, 20 (91%) of these were classified within the 2–4 DLB signs group (Table 1).

### Psychotropic medication

We found the usage of any psychotropic medication in 86% of residents. All four psychotropics (N05A, N05B, N05C and N06A) were found in 6%, three in 17%, two in 31%, and one in 32% of residents. We found the usage of anxiolytics in 58%, hypnotics/sedatives in 41%, anti-depressants in 50% (Table 2). In addition, anti-dementia medication in 33% of residents (Table 3).

**Fig. 1:**
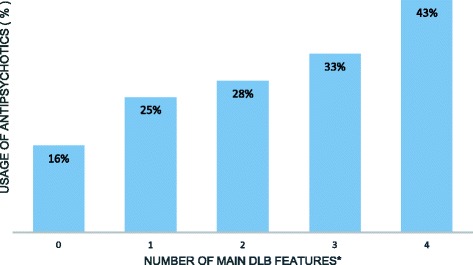
Antipsychotic treatment in relation to the number of clinical DLB features. *The total number of residents with clinical features of dementia with Lewy bodies (DLB) and antipsychotic treatment. The DLB signs are: visual hallucinations, parkinsonism, fluctuating cognition, and rapid eye movement sleep behaviour disorder. *P*-value < 0.001

Usage of antipsychotics was found in 22% of the study population, most prevalently risperidone (10%) followed by haloperidol (7%) and other atypical antipsychotics (clozapine, olanzapine and quetiapine) (2%) (Table 2). There was no difference in antipsychotic treatment according to sex or dementia diagnosis (yes/no). However, the age groups showed differences where younger residents (≤85 years) had significantly more frequent usage of antipsychotics compared with those ≥86 years (Tables 2 and 3). Furthermore, the usage of antipsychotics increased with the increasing number of main DLB signs from 25% to 43% (Fig. 1). Comparing all residents, those in the 2–4 DLB group had the highest usage of any antipsychotics (32%) and the highest usage of haloperidol (14%) (Table 2).

Of the participants with a dementia diagnosis, 45% had treatment with some anti-dementia medication. None of the residents without a dementia diagnosis had anti-dementia medication (Table 3). Younger residents (aged ≤85 years) had significantly higher usage of some anti-dementia medication (43%) compared with those aged ≥86 years (26%) (Table 3).

Comparing the 0–1 DLB and 2–4 DLB groups, there were no differences in treatment with any cholinesterase inhibitors (ChEI). However, higher usage of memantine was seen in the 2–4 DLB group (Table 3). In the group with 2–4 DLB signs, 37% were undergoing anti-dementia treatment, while 62% of those already with a diagnosis for DLB/PDD were undergoing anti-dementia treatment.

## Discussion

In this study, geographically covering all 40 NHs in the third largest city in Sweden, we found an increasing usage of antipsychotics with the increasing number of DLB signs. Furthermore, residents with two or more main clinical DLB features were also receiving more antipsychotics and less anti-dementia medication compared with the rest of the NH residents. Finally, almost 40% of the residents with 2–4 DLB signs had either dementia NOS or no dementia diagnosis, thereby being at risk for unsuitable treatment.

### Study population

A number of studies have emphasized the importance of identifying DLB/PDD patients at an early stage, which in turn may reduce the risk of an incorrect diagnosis, unsuitable medication, worsening of the quality of life, early NH admission and increased risk of shorter survival [33–37]. By dividing the study population into two groups (0–1 and 2–4 DLB signs), we could extract the high-risk residents for DLB/PDD diagnosis together with their clinical features, medical usage and actual diagnoses. The prevalence of NH residents with 2–4 DLB signs in this study was 16%. Although not specifically addressed here, the observational data from clinical nurses and recorded clinical signs at the time of the study suggest that residents with 2–4 DLB signs could have undiagnosed DLB/PDD. Furthermore, over 90% of the residents already with a diagnosis for DLB/PDD from hospital medical records were in the group with 2–4 DLB signs, which indicates that the study questionnaire was efficient in identifying residents with possible DLB/PDD (Table 1).

By using the Swedish NMDS and the hospital medical records, together with the actual clinical features reported by nursing personnel who know the residents best, we are able to present accurate medical usage for this large study population and contribute to the quality of the analysed data. Furthermore, the dementia diagnoses were investigated by resident physicians who must follow basal guidelines for dementia investigation from the Swedish NBHW [30].

Nevertheless, the study had a number of limitations. It was not possible to investigate the severity of dementia of residents due to the lack of this information. Since severe dementia according to ICD-10 is defined as a need of permanent support and caregiving from others, all residents of this study were classified as having severe dementia. Another limitation was the lack of information about why 170 elderly residents were “undiagnosed” NH dementia residents. We assume that these undiagnosed residents either were under basal dementia investigation or did not have a dementia diagnosis that was registered at the time of our data collection.

### Psychotropic medication

The usage of psychotropics is still high and according to several Swedish, Danish and Norwegian studies varies greatly (39%–71%) due to whether the elderly are institutionalized or not and managed by primary care or specialist clinics [25, 27, 28]. In our study, we show that antipsychotics, anxiolytics, hypnotic/sedatives and anti-depressants were used in the NHs to the same degree as the above mentioned with no difference by sex or dementia diagnosis (Table 2).

By dichotomizing the residents into two age groups (≤85 and ≥86 years), we showed that those ≤85 years were more frequently treated with antipsychotics (both haloperidol and risperidone), which may be appropriate according to national recommendations for treatment.

#### Antipsychotics

Large studies on conventional antipsychotics have shown an association with increased mortality risk in residents with dementia [15, 23], with additionally a high prevalence of adverse effects [38–40] such as sedation, delirium, cognitive impairment, increased risk of CVI and extra-pyramidal symptoms [11, 41]. There have been some changing trends in antipsychotic treatments over the past decade, but still with minor results [24, 42]. In our study population, risperidone was the most common antipsychotic used in residents with dementia (11,5%) followed by haloperidol (6,6%); this could be considered a good treatment policy. However, the relatively large usage of haloperidol (7,1%) and the low percentage of quetiapine and olanzapine (1,9%) are not in line with recommendations by either the Swedish NBHW, the EMA or the FDA [21, 22, 43]. For example, in a similar population in US NHs, haloperidol treatment was 1,9% and in a study from Sydney, Australia, the usage of conventional antipsychotics was 7,4% [44, 45]. Snowdon compared psychotropic medication use in NHs and noted frequencies of antipsychotics 42,6% (Finland), 23,8% (Norway), 28,0% (Australia) compared to our 21,6% [45].

As noted, individuals with DLB may have high hypersensitivity to antipsychotics with an increased risk of adverse events such as somnolence, falls, extra-pyramidal symptoms, malignant neuroleptic syndrome and finally increased mortality [13, 16, 34]. In our study, the residents with 2–4 DLB signs received the highest proportion of antipsychotics (32%) as well as the highest usage of haloperidol (17%) (Table 2).

There is currently no consensus on evidence-based treatment of NPS in DLB patients either in their homes or in NHs. However, clinical guidelines show beneficial effects from ChEI (donepezil and rivastagmin) and less beneficial effects with both atypical (quetiapine and risperidon) and conventional antipsychotics (haloperidol) [38–40]. Alarmingly, the antipsychotic usage in our study increased with the greater number of DLB clinical signs to as high as 43%, meaning that the most fragile elderly residents had the poorest treatment (Fig. 1). If we assume that these elderly persons have a possible DLB disorder, then all antipsychotic drugs would be highly inappropriate.

We found that antipsychotic treatment varied between 26% and 28% in residents with clinical features such as PD, rigidity, balance problems, excessive daytime sleepiness and sleep disorder as well as in 38% of those with visual hallucinations. One disadvantage of our study was the lack of opportunity to perform individual clinical examinations in order to observe, for example, potential extra-pyramidal symptoms due to usage of antipsychotic medication. This was however not practically possible to carry out. The reports from nursing staff is of a more longitudinal character in relation to a doctor’s examination. Against this background, the limitation must be considered as minor.

The association between drug use and the number of DLB signs is difficult to establish because the potential side effects of antipsychotics are identical to DLB signs, which represents an important limitation. However, one favourable finding is that those residents with no or one DLB sign had the highest haloperidol doses (1.5 mg–2.0 mg) compared with those with 2–4 DLB signs. This may indicate that the symptoms in the 2–4 DLB group are less influenced by antipsychotic side effects.

#### Other psychotropics

We found that the usage of psychotropics in 78% of residents was equally distributed between men and women. Comparison of the treatment of our NH residents with studies from geographically close countries showed similarities in treatment. In a study of Norwegian NHs, 88% of the elderly residents with dementia had some psychotropic medication [7]. The residents in the present study received psychotropic treatments continuously and over two-thirds had at least one anxiolytic medication, most often oxazepam. NPS such as anxiety, agitation and sleep disturbance are common in the elderly and one explanation for this extensive anxiolytic treatment might be the lower prevalence of adverse events compared with other psychotropics and antipsychotic medications.

Analysis of our two age groups showed that even the older, more fragile individuals (≥86 years) had the same amount of anxiolytic medication and hypnotics/sedatives as the younger group (≤85 years). According to the Swedish NBHW and the EMA, the prescription of inappropriate medications including psychotropics is not recommended especially for the elderly with dementia [21, 43]. However, a small positive finding from our study was that at least the usage of antipsychotics was higher in the younger and hopefully less fragile residents.

#### Anti-dementia medication

We found anti-dementia medication in 45% of the residents but in none of the residents without a formal dementia diagnosis, which may indicate that the diagnoses from hospital medical records are reliable (Table 3). In our study, there was a difference according to age in anti-dementia treatment showing that younger residents (≤85 years) had more anti-dementia medication compared with those ≥86 years. In some respects, this situation may be disadvantageous because the older population with dementia can also have good short-term cognitive response to ChEI as well as positive long-term effects [46].

Although ChEI are not the first-line medical treatment of NPS, they may reduce the emergence of NPS in the elderly with dementia and play a positive role in reducing these symptoms [47]. Of our NH residents with a formal DLB/PDD diagnosis, 62% had anti-dementia medication compared with 35% of those with 2–4 DLB signs.

In addition, one-third of the residents with 2–4 DLB clinical features were those without a diagnosis. If we assume that they have had undiagnosed dementia since becoming NH residents, having no formal dementia diagnosis may constitute a disadvantage for these elderly residents in that they miss out on anti-dementia treatment and risk treatment with inappropriate antipsychotics.

Other minor limitations, beside lack of clinical examinations, were that CT/MRI findings and family data were not available. However, observations from the clinical nurses who recorded the clinical features at the time of study suggest that residents with 2–4 DLB signs should be reported to the NH physicians as high-risk individuals for undiagnosed DLB/PDD and are subsequently unsuited for treatment with psychotropics such as haloperidol and less beneficial anti-dementia treatments.

Nowadays, Swedish NHs may have 30–100 residents with one resident physician responsible for the medical treatment of all these individuals, which implies that collaboration with the nursing staff is crucial. For example, the NH staff continuously report different medical signs of importance to guide the active physicians working at a NH to consider different diagnoses. Our study questionnaire was not standardised which may seem as a limitation. However, it was based on the main DLB symptoms described in the consensus criteria. By using the clinical DLB signs from our study questionnaire as an observation manual for NH staff, we aimed to identify residents with two or more DLB signs and categorize them as at-risk individuals for inappropriate medication who could be reported to the physicians for further treatment or investigation.

## Conclusions

Residents from Swedish NHs with 2–4 clinical DLB signs receive an unfavourable medical treatment with high antipsychotic usage and insufficient anti-dementia medication. These findings show the importance of identifying elderly persons with DLB features more effectively and improving collaboration with nursing staff to provide better medical prescription.

## Abbreviations

AD: Alzheimer’s disease
AD-MIX: Alzheimer’s disease mixed dementia
CBCT: Cone beam computed tomography
DLB: Dementia with Lewy bodies
EMA: European Medicines Agency
FDA: Food and Drug Administration
MR: Magnetic resonance
NBHW: National Board of Health and Welfare
NH: Nursing home
NOS: Not otherwise specified
NPS: Neuropsychiatric symptoms
PDD: Parkinson’s disease with dementia
RBD: Sleep behaviour disorder
REM: Rapid eye movement
VaD: Vascular dementia
